# Detection of *Anaplasma phagocytophilum*, *Babesia microti*, *Borrelia burgdorferi*, *Borrelia miyamotoi*, and Powassan Virus in Ticks by a Multiplex Real-Time Reverse Transcription-PCR Assay

**DOI:** 10.1128/mSphere.00151-17

**Published:** 2017-04-19

**Authors:** Rafal Tokarz, Teresa Tagliafierro, D. Moses Cucura, Ilia Rochlin, Stephen Sameroff, W. Ian Lipkin

**Affiliations:** a Center for Infection and Immunity, Mailman School of Public Health, Columbia University, New York, New York, USA; b Division of Vector Control, Suffolk County Department of Public Works, Yaphank, New York, USA; University of Wisconsin—Madison

**Keywords:** *Borrelia burgdorferi*, babesiosis, ticks

## Abstract

*Ixodes scapularis* ticks are implicated in transmission of *Anaplasma phagocytophilum*, *Borrelia burgdorferi*, *Borrelia miyamotoi*, *Babesia microti*, and Powassan virus. We describe the establishment and implementation of the first multiplex real-time PCR assay with the capability to simultaneously detect and differentiate all five pathogens in a single reaction. The application of this assay for analysis of ticks at sites in New York and Connecticut revealed a high prevalence of *B. microti* in ticks from Suffolk County, NY. These findings are consistent with reports of a higher incidence of babesiosis from clinicians managing the care of patients with tick-borne diseases in this region.

**IMPORTANCE** The understanding of pathogen prevalence is an important factor in the determination of human risks for tick-borne diseases and can help guide diagnosis and treatment. The implementation of our assay addresses a critical need in surveillance of tick-borne diseases, through generation of a comprehensive assessment of pathogen prevalence in *I. scapularis*. Our finding of a high frequency of ticks infected with *Babesia microti* in Suffolk County, NY, implicates this agent as a probable frequent cause of non-Lyme tick-borne disease in this area.

## Abstract (precis)

The understanding of pathogen prevalence is an important factor in the determination of human risks for tick-borne diseases and can help guide diagnosis and treatment. The implementation of our assay addresses a critical need in surveillance of tick-borne diseases, through generation of a comprehensive assessment of pathogen prevalence in *I. scapularis*. Our finding of a high frequency of ticks infected with *Babesia microti* in Suffolk County, NY, implicates this agent as a probable frequent cause of non-Lyme tick-borne disease in this area.

## OBSERVATION

Tick-borne diseases (TBD) are the most common vector-borne diseases in the United States (1). Lyme disease alone accounts for an estimated 300,000 annual cases of TBD (2). Of the approximately 90 tick species distributed throughout the continental United States, *Ixodes scapularis* is the species most frequently associated with human disease. *I. scapularis* is the primary vector of *Borrelia burgdorferi*, the causative agent of Lyme disease, *Anaplasma phagocytophilum*, the agent of human granulocytic anaplasmosis, *Babesia microti*, the agent of babesiosis, *Borrelia miyamotoi*, and the deer tick virus clade of Powassan virus (POWV) (3). The surveillance of tick-borne pathogens is not well developed despite the fact that these diseases are underreported and are undergoing rapid range expansion (4, 5). In many areas where TBD are endemic, all agents cocirculate, increasing the likelihood of coinfection of both ticks and humans (6–9). Established multiplex real-time PCR assays typically target *B. burgdorferi*, *B. microti*, and *A. phagocytophilum*, but not *B. miyamotoi* or POWV (10–12). To enable surveillance for all major *I. scapularis*-borne pathogens, we developed a multiplex one-step real-time reverse transcription-PCR (RT-PCR) assay that targets *A. phagocytophilum*, *B. microti*, *B. miyamotoi*, *B. burgdorferi*, and POWV, and we employed this assay to test ticks from areas where TBD are highly endemic in the northeastern United States.

Multiple-sequence alignments of each selected gene target were used to identify loci for primer and probe design (Table 1). *ospA*, a plasmid-borne gene exclusive to Lyme borreliosis-*Borrelia* was selected as a target for *B. burgdorferi*. For *B. miyamotoi*, we exploited the high sequence variability of the flagellar *flaB* gene within *Borrelia* to select *B. miyamotoi*-specific primer/probe sequences. The mitochondrial *cox-1* gene and the 16S rRNA gene were selected as targets for *B. microti* and *A. phagocytophilum*, respectively. For POWV (lineage II-deer tick virus), we selected a conserved sequence within the 3′ untranslated region. Multiple primer/probe sets were designed for each pathogen and tested on known pathogen-positive tick samples to identify the optimal primer/probe combination. Quantified plasmid standards were generated for every agent by amplification and cloning of genomic regions encompassing the primer/probe binding sites. DNA standards were quantified by using a Qubit DNA high-sensitivity assay kit (Life Technologies, Inc.) and serially diluted in a DNA background (100 ng/μl). For POWV, we generated *in vitro*-transcribed RNA standards using the RiboMax large-scale RNA production system kit (Promega). The POWV standards were quantified using the Qubit RNA high-sensitivity assay kit (Life Technologies, Inc.) and serially diluted in background RNA (100 ng/μl). The primer/probe sets were selected based on their performance in individual and multiplex PCRs with quantified DNA and RNA standards and total nucleic acid (TNA) derived from pathogen-positive ticks as the templates. Sensitivity in a multiplex reaction was experimentally measured to be ≥5 DNA copies for each DNA agent and ≥10 RNA copies for POWV. Sensitivity was not altered for any primer pair for multiplex versus single-agent PCRs. To examine the capacity of the assay to simultaneously detect >1 agent, we tested samples spiked with multiple serially diluted standards (see Table S1 in the supplemental material). In assays with three and four standards, all agents were detected when present at 10 copies, irrespective of the agent tested. In assays of standards representing five agents, *A. phagocytophilum*, *B. microti*, *B. burgdorferi*, and *B. miyamotoi* were all detected at 10^1^ copies; POWV was detected at 20 RNA copies. No amplification products were observed in assays of *Dermacentor variabilis* or *Amblyomma americanum* ticks, which do not carry these agents.

10.1128/mSphere.00151-17.2 TABLE S1  Multiplex assay sensitivity in detection of multiple agents. Download TABLE S1, PDF file, 0.05 MB. Copyright © 2017 Tokarz et al. 2017 Tokarz et al. This content is distributed under the terms of the Creative Commons Attribution 4.0 International license.

**TABLE 1:** Primer and probe sequences

Agent	Gene target	Forward primer	Reverse primer	Probe^a^
*Borrelia burgdorferi*	*ospA*	CCTTCAAGTACTCCAGATCCATTG	AACAAAGACGGCAAGTACGATC	FAM-CAACAGTAGACAAGCTTGA-MGB
*Borrelia miyamotoi*	*flaB*	AGCACAAGCTTCATGGACATTGA	GAGCTGCTTGAGCACCTTCTC	VIC-TGTGGGTGCAAATCAGGATGAAGCA-BHQ
*Babesia microti*	*cox1*	CATCATGCCAGGCCTGTTTG	GAAGAAACCACAAGAGCAAATGC	Quasar 705-TACTACCCATACTGGTCGGTGCTCC-BHQ
*Anaplasma phagocytophilum*	16S rRNA	GGCATGTAGGCGGTTCGGT	CACTAGGAATTCCGCTATCCTCTCC	Cy5-GCCAGGGCTTAACCCTGGAGCT-BHQ
Powassan virus	3′-UTR	GTGATGTGGCAGCGCACC	CTGCGTCGGGAGCGACCA	Texas Red-CCTACTGCGGCAGCACACACAGTG-BHQ

a Abbreviations: FAM, 6-carboxyfluorescein; MGB, minor groove binder; BHQ, black hole quencher dye.

*I. scapularis* ticks were collected in 2015 and 2016 by tick dragging at five sites in Suffolk County, NY (Southampton, Mannorville, Southold, Islip, and Huntington) and three sites in Connecticut (Mansfield in Tolland County and Stamford and Greenwich in Fairfield County) (Fig. S1). Ticks (89 adults and 115 nymphs from New York and 86 adults and 28 nymphs from Connecticut) were homogenized in phosphate-buffered saline prior to nucleic acid extraction (Easy Mag extraction platform; BioMérieux). After control for DNA and RNA integrity, total nucleic acid was subjected to a one-step RT-PCR performed on a Bio-Rad C1000 Touch system with a CFX96 optical module using the Invitrogen RNA UltraSense one-step quantitative RT-PCR system. The reverse transcription step was performed at 55°C for 15 min followed by incubation at 95°C for 10 min. The PCR consisted of 40 cycles (95°C for 15 s and 60°C for 30 s). All tick samples were tested twice, in assays performed on different days. Assay specificity was confirmed by retesting positive samples by PCR using alternate primer pairs and dideoxy sequencing of PCR products.

10.1128/mSphere.00151-17.1 FIG S1  Tick collection sites. Sites 1 to 5 represent Huntington, Islip, Mannorville, Southold, and Southampton in Suffolk County, NY. Sites 6 to 8 represent Stamford, Greenwich, and Mansfield in Connecticut. Filled circles indicate sites of nymph collections; an x represents a site of adult tick collection. The map was generated using StepMap. Download FIG S1, PDF file, 0.2 MB. Copyright © 2017 Tokarz et al. 2017 Tokarz et al. This content is distributed under the terms of the Creative Commons Attribution 4.0 International license.

*B. burgdorferi* was the most frequently detected agent in ticks from Suffolk County, with 21% of nymphs and 67% of adults ticks infected (Table 2). *B. microti* was the second most prevalent agent, present in 17% of nymphs and 30% of adults. *A. phagocytophilum* was present in 7% of nymphs and 11% of adults. POWV was detected in 2% of adults, and *B. miyamotoi* was present in 3% of both nymphs and adults. Coinfections were detected in 11% of nymphs and 45% of adults, including one nymph and seven adults (4%) that were positive for *A. phagocytophilum*, *B. burgdorferi*, and *B. microti* and one adult tick that was positive for *A. phagocytophilum*, *B. burgdorferi*, and* B. miyamotoi*.

**TABLE 2:** Tick screening results

State, stage, and site (*n*)	No. (%) of tick forms positive for:					Coinfecting organisms detected [no. (%)]
	*A. phagocytophilum*	*B. burgdorferi*	*B. microti*	*B. miyamotoi*	Powassan virus	
New York nymphs						*B. burgdorferi*/*B. microti*: 6 (5); *B. burgdorferi*/*A. phagocytophilum*: 4 (3); *B. burgdorferi*/ *B. microti*/*A. phagocytophilum*: 1 (<1); uninfected: 71 (62)
Tuckahoe, Southampton (38)	2 (5)	9 (24)	7 (18)	1 (<3)	0	
Mannorville (62)	5 (8)	11 (18)	6 (10)	3 (5)	0	
Laurel Lake, Southold (15)	1 (7)	4 (27)	6 (40)	0	0	
Total NY nymphs (115)	8 (7)	24 (21)	19 (17)	4 (3)	0	
New York adult ticks						*B. burgdorferi*/*B. microti*: 19 (21); *B. burgdorferi*/*A. phagocytophilum*: 10 (11); *B. burgdorferi*/*B. miyamotoi*: 3 (3); *B. microti*/*A. phagocytophilum*: 3 (2); *B. burgdorferi*/ Powassan virus: 2 (2); *B. burgdorferi*/*B. microti*/*A. phagocytophilum*: 7 (8); *B. burgdorferi*/*B. microti*/*B. miyamotoi*: 1 (1); uninfected: 23 (26)
Connetquot State Park, Islip (43)	7 (16)	32 (74)	15 (35)	2 (5)	0	
Caumsett State Park, Huntington (46)	3 (7)	28 (61)	12 (26)	1 (<2)	2 (2)	
Total NY adults (89)	10 (11)	60 (67)	27 (30)	3 (3)	2 (2)	
Connecticut nymphs						*B. burgdorferi/B. microti*: 1 (4); *B. burgdorferi*/*A. phagocytophilium*: 1 (4)
Mansfield (28)	2 (7)	7 (25)	2 (7)	0	0	
Connecticut adults						*B. burgdorferi/B. microti*: 10 (12); *B. burgdorferi*/*B. miyamotoi*: 1 (1); *B. burgdorferi*/*A. phagocytophilium*: 5 (6); *B. burgdorferi*/Powassan virus: 1 (2);* B. burgdorferi*/*B. microti*/*B. miyamotoi*: 1 (2); uninfected: 28 (32)
Mianus River Park, Stamford (21)	1 (5)	14 (66)	3 (14)	1 (5)	1 (5)	
Babcock preserve, Greenwich (65)	6 (9)	39 (60)	10 (15)	2 (5)	0	
Total CT adults (86)	7 (8)	53 (62)	13 (15)	3 (3)	1 (1)	

The prevalence of *B. burgdorferi* was similar in Connecticut (in 25% of nymphs and 62% of adult ticks). Although *B. microti* was the second most prevalent agent, only 7% of nymphs and 14% of adults were infected. *A. phagocytophilum* was present in 7% of nymphs and 8% of adults. *B. miyamotoi*-infected (3 positive adults) and POWV-infected ticks (1 positive adult) were rare. Only one tick was positive for three agents (*B. burgdorferi*, *B. microti*, and *B. miyamotoi*).

An important benefit of this multiplex assay is the capacity to test for *B. miyamotoi* and POWV, two agents rarely included in tick-borne pathogen surveillance studies. *B. miyamotoi* has only recently been implicated in TBD, and many of its clinical and epidemiological aspects are still not well understood (13). For POWV, improvement in surveillance is essential in light of the potential for POWV to result in life-threatening encephalitis (14). Based on the infrequent numbers of reported human cases, the prevalence of *B. miyamotoi* and POWV are presumed to be low relative to other *I. scapularis*-transmitted agents. This was corroborated with the results of our assay, where neither pathogen was present at >5% prevalence at any site or life stage tested.

We detected a significantly higher prevalence of *B. microti* in adults from Suffolk County than at sites in Connecticut (*P* = 0.019). The prevalence of *B. microti* in nymphs was also higher in Suffolk County, but the difference was not significant. In prior studies of *I. scapularis* nymphs throughout New York, the prevalence of *B. microti* ranged from 3% to 12% (6, 15). The *B. microti* infection rate in nymphs from Suffolk County (17%) was higher and similar to the percentage of ticks positive for *B. burgdorferi* (21%). We also found 30% of adult ticks positive for *B. microti*, and this was among the highest reported prevalence rates for *B. microti* in *I. scapularis* adults (16). In contrast, the lower prevalence of *B. microti* in ticks collected in Fairfield and Tolland Counties in Connecticut more closely reflects *B. microti* infection rates from areas where Lyme disease is endemic. This discrepancy reflects the frequency of babesiosis in the areas surveyed; in 2014, 197 babesiosis cases were reported in Suffolk County alone, compared to 212 in the entire state of Connecticut (1; http://www.health.ny.gov/statistics/diseases/communicable/2014/docs/cases.pdf).

In 2016, 3,199 cases of Lyme disease and 327 cases of babesiosis were reported in New York (https://data.cdc.gov/NNDSS/NNDSS-Table-II-Babesiosis-to-Campylobacteriosis/4y34-2pku; https://data.cdc.gov/NNDSS/NNDSS-Table-II-Lyme-disease-to-Meningococcal/93k9-hy54). This near-10-to-1 ratio of Lyme disease to babesiosis has been a consistent trend in New York in recent years. In Suffolk County, a region with New York state’s highest percentage of babesiosis, this ratio declines to approximately 2 to 1 (http://www.health.ny.gov/statistics/diseases/communicable/2015/docs/cases.pdf). Although factors such as physician awareness and enhanced testing may contribute to this disparity, our data suggest that it may reflect a higher prevalence of *B. microti*-infected ticks present in Suffolk County. Rates of coinfections may also be higher; 25% of *B. burgdorferi*-positive nymphs were also positive for *B. microti*. A recent study by Curcio et al. indicated that 29% of sera from Lyme disease patients also tested positive for antibodies to *B. microti*, although it was unclear if the infections were concurrent (17). The importance of this finding is underscored by the fact that antibiotics against Lyme disease have no impact on the clinical course of babesiosis. Surveillance studies in ticks and testing in clinical microbiology laboratories to address coinfections may have implications for management of TBD and public health.
