# Supplementary material for: Detection of Anaplasma phagocytophilum, Babesia microti, Borrelia burgdorferi, Borrelia miyamotoi, and Powassan Virus in Ticks by a Multiplex Real-Time Reverse Transcription-PCR Assay
Source: mSphere. 2017 Apr 19;2(2):e00151-17. doi: 10.1128/mSphere.00151-17 (PMC5397568; doi:10.1128/mSphere.00151-17)
Supplement: TABLE S1 [file sph002172269st2.pdf]

Supplemental Table 1. Multiplex assay sensitivity in detection of multiple agents

3 agents present		4 agents present		5 agents present	
Agent	Sensitivity (copies)	Agent	Sensitivity (copies)	Agent	Sensitivity (copies)
A. phagocytophilum	10	*A. phagocytophilum*	10	*A. phagocytophilum*	10
B. microti	10	*B. microti*	10	*B. microti*	10
B. burgdorferi	10	*B. burgdorferi*	10	*B. burgdorferi*	10
B. miyamotoi	10	*B. miyamotoi*	10	*B. miyamotoi*	10
Powassan virus	10	Powassan virus	10	Powassan virus	20

In assays with 3 and 4 standards, different combinations of agents were tested with equal sensitivity results.

Concentrations tested included 10, 20, 50, 100 and 1000 copies present at equal concentration for each agent.
