# Supplementary figures and images for: Detection of Anaplasma phagocytophilum, Babesia microti, Borrelia burgdorferi, Borrelia miyamotoi, and Powassan Virus in Ticks by a Multiplex Real-Time Reverse Transcription-PCR Assay
Source: mSphere. 2017 Apr 19;2(2):e00151-17. doi: 10.1128/mSphere.00151-17 (PMC5397568; doi:10.1128/mSphere.00151-17)

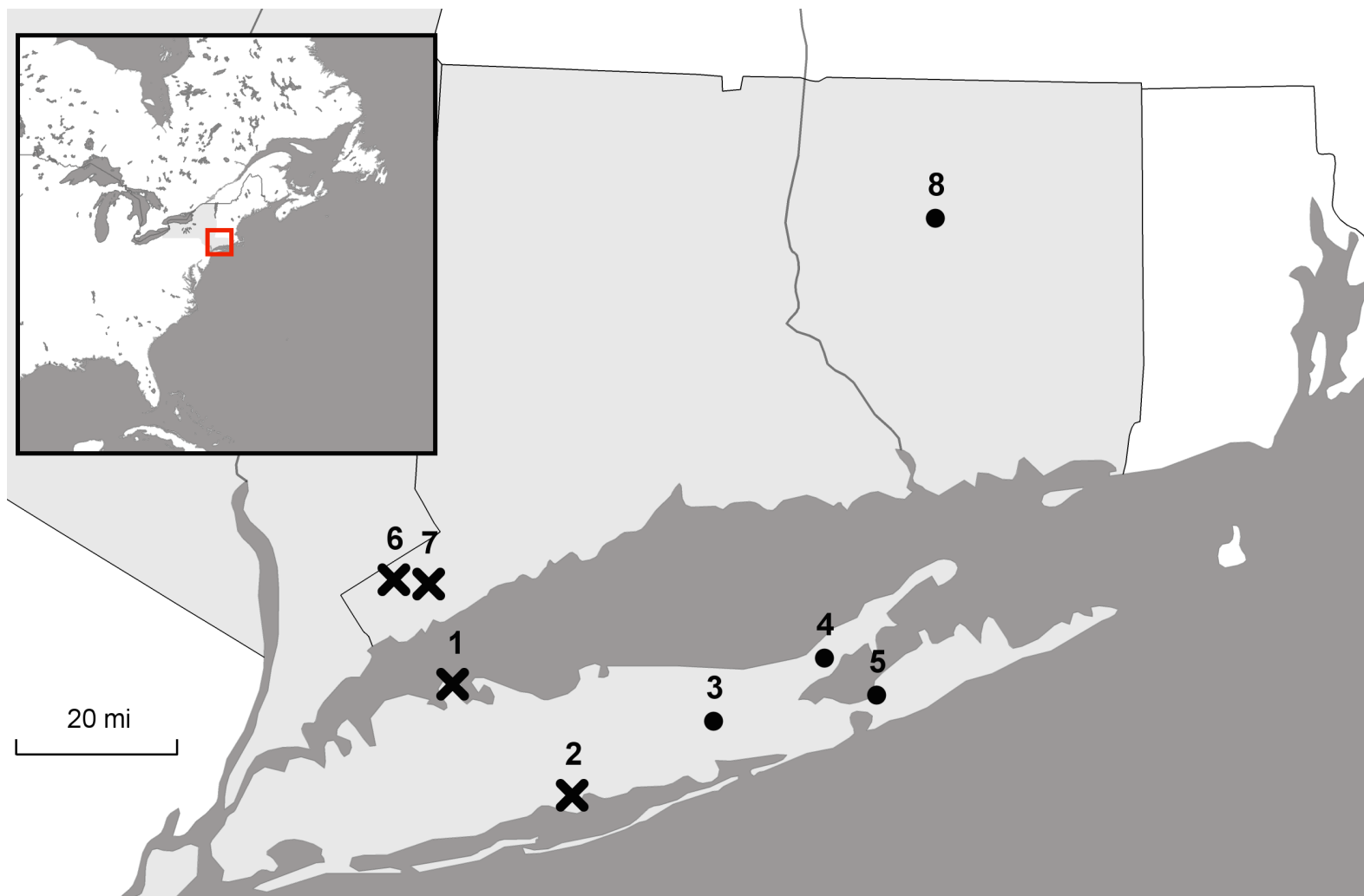

Supplement: FIG S1 [file sph002172269sf1.pdf]
